# EFFECT: a randomized phase II study of efficacy and impact on function of two doses of nab-paclitaxel as first-line treatment in older women with advanced breast cancer

**DOI:** 10.1186/s13058-020-01319-1

**Published:** 2020-08-05

**Authors:** Laura Biganzoli, Saverio Cinieri, Rossana Berardi, Rebecca Pedersini, Amelia McCartney, Alessandro Marco Minisini, Elena Rota Caremoli, Simon Spazzapan, Emanuela Magnolfi, Antonella Brunello, Emanuela Risi, Raffaella Palumbo, Silvana Leo, Marco Colleoni, Sara Donati, Sabino De Placido, Laura Orlando, Mirco Pistelli, Veronica Parolin, Anna Mislang, Dimitri Becheri, Fabio Puglisi, Giuseppina Sanna, Elena Zafarana, Luca Boni, Giuseppe Mottino

**Affiliations:** 1 “Sandro Pitigliani” Department of Medical Oncology, Hospital of Prato, ASL Toscana Centro, Prato, Italy; 2 Department of Medical Oncology, Perrino Hospital, ASL Brindisi, Brindisi, Italy; 3 Department of Medical Oncology, Ospedali Riuniti di Ancona, Università Politecnica delle Marche, Ancona, Italy; 4 Breast Oncology Unit, Hospital Civili di Brescia, Brescia, Italy; 5 Department of Oncology, Azienda Ospedaliero Universitaria Integrata di Udine, Udine, Italy; 6 Cancer Centre, Azienda Socio Sanitaria Territoriale Papa Giovanni XXIII, Bergamo, Italy; 7 Unit of Medical Oncology and Cancer Prevention, IRCCS CRO di Aviano, National Cancer Institute, Aviano, Italy; 8 Department of Medical Oncology, Hospital Civile SS Trinità di Sora, Frosinone, Italy; 9 Department of Medical Oncology, Veneto Institute of Oncology IOV Padova, Padua, Italy; 10 Department of Medical Oncology, IRCCS ICS Maugeri, Pavia, Italy; 11 Department of Medical Oncology, Vito Fazzi Hospital, Lecce, Italy; 12 Division of Medical Senology, IEO, European Institute of Oncology IRCCS, Milan, Italy; 13 Department of Oncology, Versilia Hospital (Camaiore-Lu), Viareggio, Italy; 14 Department of Endocrinology and Molecular and Clinical Oncology, AOU Federico II, Naples, Italy; 15 Department of Medical Oncology, Azienda Ospedaliera Universitaria Integrata, Verona, Italy; 16 Flinders Centre for Innovation in Cancer, Flinders Medical Centre, Bedford Park, South Australia Australia; 17 Geriatric Medicine Unit, AUSL Toscana Centro, Prato, Italy; 18 Clinical Trials Centre, AOU University Hospital Careggi, Florence, Italy

**Keywords:** Nab-paclitaxel, Functional decline, Toxicity, Breast cancer, Older adults, Metastatic

## Abstract

**Background:**

Limited data are available regarding the use of nab-paclitaxel in older patients with breast cancer. A weekly schedule is recommended, but there is a paucity of evidence regarding the optimal dose. We evaluated the efficacy of two different doses of weekly nab-paclitaxel, with a specific focus on their corresponding impact on patient function, in order to address the lack of data specifically relating to the older population.

**Methods:**

EFFECT is an open-label, phase II trial wherein 160 women with advanced breast cancer aged ≥ 65 years were enrolled from 15 institutions within Italy. Patients were randomly assigned 1:1 to receive nab-paclitaxel 100 mg/m^2^ (arm A) or 125 mg/m^2^ (arm B) on days 1, 8, and 15 on a 28-day cycle, as first-line treatment for advanced disease. The primary endpoint was event-free survival (EFS), wherein an event was defined as disease progression (PD), functional decline (FD), or death. In each arm, the null hypothesis that the median EFS would be ≤ 7 months was tested against a one-sided alternative according to the Brookmeyer Crowley test. Secondary endpoints included objective response rate (ORR), clinical benefit rate (CBR), progression-free survival (PFS), overall survival (OS), and safety.

**Results:**

After a median follow-up of 32.6 months, 140 events were observed in 158 evaluable patients. Median EFS was 8.2 months (90% CI, 5.9–8.9; *p* = 0.188) in arm A vs 8.3 months (90% CI, 6.2–9.7, *p* = 0.078) in arm B. Progression-free survival, overall survival, and response rates were similar in both groups. A higher percentage of dose reductions and discontinuations due to adverse events (AEs) was noted in arm B. The most frequently reported non-haematological AEs were fatigue (grade [G] 2–3 toxicity occurrence in arm A vs B, 43% and 51%, respectively) and peripheral neuropathy (G2–3 arm A vs B, 19% and 38%, respectively).

**Conclusion:**

Pre-specified outcomes were similar in both treatment arms. However, 100 mg/m^2^ was significantly better tolerated with fewer neurotoxicity-related events, representing a more feasible dose to be recommended for older patients with advanced disease.

**Trial registration:**

EudraCT, 2012-002707-18. Registered on June 4, 2012. NIH ClinicalTrials.gov, NCT02783222. Retrospectively registered on May 26, 2016.

## Background

Older patients are at higher risk of chemotherapy-related toxicities in comparison with younger adults [1]. Weekly solvent-based taxanes, such as paclitaxel and docetaxel, are amongst the recommended agents to treat older patients with advanced breast cancer (ABC) [2]. However, close monitoring is required given that treatment-induced side effects, particularly neurotoxicity and fatigue, place older patients at risk of subsequent functional decline (FD) [3–7]. Nanoparticle albumin-bound paclitaxel (nab-paclitaxel) does not require steroid premedication and is associated with a lower rate of hypersensitivity reactions [8–10], thus representing an efficacious and safe alternative to solvent-based taxanes. Additionally, recovery from nab-paclitaxel-induced neurotoxicity is reputedly shorter than with solvent-based taxanes [8, 9], which may subsequently produce a reduction in negative functional impact. Limited data exist regarding the use of nab-paclitaxel in elderly patients, and uncertainty still prevails regarding the ideal dose to be used in this population. A previous post hoc analysis of two studies investigated the safety and efficacy of q1w and q3w nab-paclitaxel compared with q3w solvent-based paclitaxel and docetaxel in older patients with ABC [11]. Two doses of weekly nab-paclitaxel (150 mg/m^2^ and 100 mg/m^2^, on days 1, 8, and 15 on a 28-day cycle) were evaluated. Nab-paclitaxel was found to be safer and more efficacious when administered weekly. However, these conclusions were limited by way of a small studied group: only 24 patients aged ≥ 65 years were treated with weekly nab-paclitaxel. Additionally, the reported 20% incidence of grade 3 sensory neuropathy was concerning.

The EFFECT trial aimed to identify the optimal weekly dose of nab-paclitaxel that could be effectively used in older patients with ABC, whilst also integrating geriatric assessment tools to evaluate the impact of treatment on function.

## Methods

### Study design and conduct

EFFECT (EudraCT 2012-002707, ClinicalTrials.gov identifier: NCT02783222) is an open-label, randomized, phase II study evaluating two doses of weekly nab-paclitaxel as the first-line treatment in older women with ABC. Patients were recruited across 15 cancer centres in Italy, with prospective approval of the protocol by local independent ethics committees at each site. Written informed consent was obtained from all patients.

### Patients and treatments

Eligible patients were female aged ≥ 65 years with pathologically confirmed ABC of any hormone receptor (HR) and HER2 status, and a history of no prior lines of treatment in the advanced setting. In line with the absence of inclusion of anti-HER2 agents in the trial protocol, women with HER2-positive disease were required to have contraindications to their administration. Additional inclusion criteria were Eastern Cooperative Oncology Group (ECOG) performance status 0–2, the presence of measurable or evaluable disease according to the Response Evaluation Criteria in Solid Tumors version 1.1 (RECIST v1.1), adequate organ function, and an absence of active/symptomatic central nervous system metastases and/or grade ≤ 1 peripheral neuropathy.

Using an interactive web response system (IWRS) and the minimization algorithm, patients were centrally randomized 1:1 to receive nab-paclitaxel 100 mg/m^2^ (arm A) or 125 mg/m^2^ (arm B) on days 1, 8, and 15 on a 28-day cycle. Patients were stratified according to age (65–74 vs ≥ 75 years), concomitant diabetes (yes/no), instrumental activity of daily living (IADL) impairment (yes/no), presence of any grade 3–4 illness according to the Cumulative Illness Rating Scale-Geriatric (CIRS-G) score (yes/no), measurable vs evaluable disease, treating centre, and prior exposure to taxanes (yes/no).

Dose modifications for specific treatment-emergent toxicities were mandated by the protocol. Patients continued on trial-assigned treatment until the point of FD, unacceptable toxicity, consent withdrawal, disease progression (PD), or death, or at the discretion of the treating physician.

### Study endpoints

The primary study endpoint was event-free survival (EFS), wherein an “event” was either FD, PD, or death. FD was defined as a decrease of at least one point from baseline values of activity of daily living (ADL) and/or instrumental activity of daily living (IADL) considered by the investigator to be treatment-related and confirmed at subsequent cycle. Secondary endpoints included objective response rate (ORR), clinical benefit rate (CBR), PFS, overall survival (OS), and safety. ORR was defined by the percentage sum of complete responses (CR) and partial responses (PR). CBR was calculated by combining CR, PR, and stable disease (SD). ORRs and CBRs were determined only in patients with measurable disease. The calculation of all time-to-event intervals started from the date of randomization. All serious and non-serious adverse events (AEs) were graded according to the Common Terminology Criteria for Adverse Events (CTCAE) version 4.03. Both events related and unrelated to the study treatment were captured.

### Assessments

Tumours were evaluated according to RECIST v1.1 within 28 days before randomization and then every 12 weeks until PD. The presence of comorbidities and functional impairments was assessed at baseline using CIRS-G [12], ADL [13], and IADL [14] instruments. ADLs and IADLs were also re-evaluated on day 1 of each cycle. AEs were recorded and graded according to CTCAE version 4.03 and were evaluated by the investigator at every patient visit from baseline until up to 30 days after discontinuation of trial treatment.

### Statistical analysis

Efficacy analyses were performed on the modified intention-to-treat population, which included all randomly assigned patients who received at least one dose of the study drug. In each arm, the null hypothesis that the median EFS would be ≤ 7 months was tested against a one-sided alternative according to the Brookmeyer Crowley test [15]. It was estimated that with the enrolment of 144 patients (72 in each arm), every hypothesis test would have a type I error rate of 5% and a power of 90% when the true median EFS was ≥ 12 months. The distributions of all studied patients according to demographic, clinical, and biologic characteristics and categorical outcomes were summarized as frequencies and percentage. Continuous variables were reported as median and range of variation. The median period of follow-up and its interquartile range were calculated for the entire study cohort according to the reverse Kaplan-Meier method. Distributions of EFS, PFS, and OS were estimated using the Kaplan-Meier product-limit method. According to the study design, no formal statistical comparisons between the results observed in the two treatment arms were planned or performed. The SAS software version 9.2 (SAS Institute, Cary, NC) was used for the statistical analysis.

During data cleaning, it was observed that some patients had two consecutive reports of G2/3 neurotoxicity or fatigue (which, by CTCAE definition, imply functional impairment of ADLs or IADLs) who were not also reported as having FD. Accordingly, a central review was undertaken. For reports of neurotoxicity, FD was confirmed and recorded in the setting of two consecutive reports of grade 2 or 3 episodes. In the setting of two consecutive reports of grade 2 or 3 fatigue, the responsible investigator was contacted, and FD was declared/confirmed only when fatigue was deemed to be treatment-related. The primary study endpoint is subsequently reported both by the investigator and by a central review.

As there is an ongoing and unresolved discussion regarding which age should be used to define “elderly” patients, an unplanned subgroup analysis was also conducted to evaluate major treatment outcomes in patients aged ≥ 75 years.

## Results

**Fig. 1:**
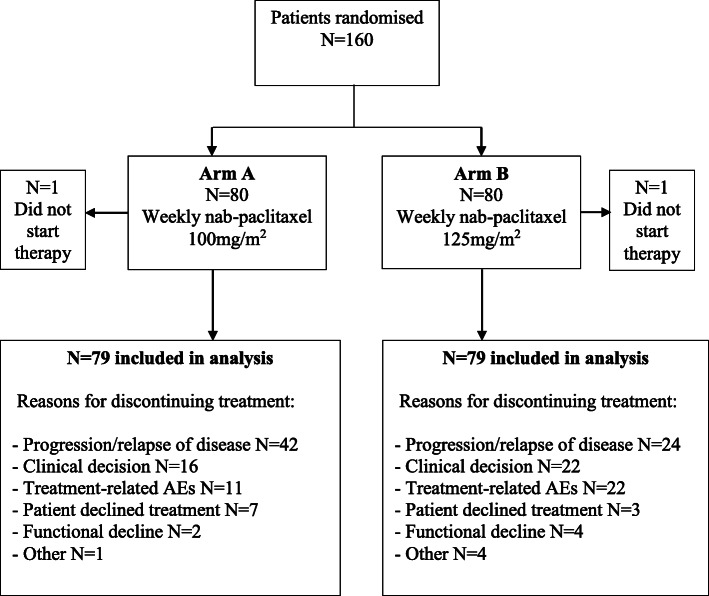
CONSORT diagram illustrating the disposition to treatment arms, plus reasons for discontinuing treatment on trial

Between January 2013 and September 2016, 160 patients were accrued and randomized in a 1:1 fashion. Overall, 79 patients per arm were evaluable for efficacy and safety analyses (see CONSORT diagram: Fig. 1). The baseline demographics are summarized in Table 1. The median age was 72 years in arm A and 73 years in arm B. Overall, approximately 70% were aged ≥ 70 years and > 40% were ≥ 75 years. The majority of patients had excellent baseline functional status and well-controlled comorbidities.

**Table 1:** Baseline characteristics of the ITT population, by treatment arm and age. Reported as *n* (%) unless otherwise indicated

Characteristics	Arm A, 100 mg/m^**2**^		Arm B, 125 mg/m^**2**^	
	Overall (***N*** = 79)	Patients aged 75+ (***N*** = 33)	Overall (***N*** = 79)	Patients aged 75+ (***N*** = 33)
**Median age in years (range)**	72 (65–84)	80 (75–84)	73 (65–88)	77 (75–88)
**Age**				
65–69	28 (35)	–	21 (27)	–
75+	33 (42)	33 (100)	33 (42)	33 (100)
**ECOG PS**				
0	51 (65)	20 (61)	43 (54)	19 (58)
1	19 (24)	9 (27)	32 (41)	13 (40)
2	9 (11)	4 (12)	4 (5)	1 (3)
**ADL scores**				
Impaired [range]	14 (18) [5/6–4/6]	6 (18) [5/6–5/6]	20 (25) [5/6–1/6]	8 (24) [5/6–1/6]
Missing data	2 (2)	2 (2)	0	0
**IADL scores**				
Impaired [range]	20 (25) [7/8–2/8]	9 (3) [7/8–4/8]	20 (25) [7/8–2/8]	8 (24) [7/8–3/8]
Missing data	2 (2)	1 (3)	0	0
**Comorbidities**				
Any grades 3–4	8 (10)	6 (18)	10 (13)	3 (9)
Diabetes mellitus	9 (11)	6 (18)	11 (14)	4 (12)
**HR status**				
ER− and PgR−	9 (11)	5 (15)	8 (10)	4 (12)
ER+ and/or PgR+	68 (86)	28 (85)	67 (85)	28 (85)
Missing data	2 (3)	0	4 (5)	1 (3)
**HER2 status**				
Positive	2 (3)	1 (3)	0	0
Missing data	5 (6)	2 (6)	10 (13)	4 (12)
**Prior taxane use**	11 (14)	3 (9)	10 (13)	1 (3)
**Measurable disease**	60 (76)	22 (67)	65 (82)	28 (85)
**Visceral disease**	56 (71)	22 (67)	55 (69)	25 (76)

*ADL* activities of daily living, *ECOG PS* Eastern Cooperative Oncology Group Performance Status, *IADL* instrumental ADL, *+* positive, *−* negative

### Treatment exposure

Overall in both arms, patients received a median of 6 cycles of nab-paclitaxel. In patients aged 75 and over, those in arm A completed a median of 6 cycles, with a median of 5 cycles completed by those assigned to arm B (Table 2). Dose delays were reported in 21% of cycles undertaken in arm A and in 23% of cycles in arm B. More than 70% of patients had dose reductions overall—however, a higher percentage of dose reductions per cycle was noted in arm B (39% vs 58%) (Table 2). The main reasons for treatment discontinuations were (arm A vs B) PD (53% vs 30%), clinical decision (20% vs 28%), AEs (14% vs 28%), patient choice (9% vs 4%), and FD (2.5% vs 5%).

**Table 2:** Treatment exposure, described by randomized arm, as well as within the subgroup aged ≥ 75. Reported as *n* (%) unless otherwise stated

Regimen received	Arm A, 100 mg/m^**2**^		Arm B, 125 mg/m^**2**^	
	All (***N*** = 79)	Pts aged 75+ (***N*** = 33)	All (***N*** = 79)	Pts aged 75+ (***N*** = 33)
Total *n* of cycles	594	214	443	180
Median *n* cycles (range)	6 (1–28)	6 (1–19)	6 (1–22)	5 (1–11)
Dose delays				
*N* of patients	52 (66)	19 (58)	50 (63)	18 (55)
*N* of cycles	127 (21)	43 (20)	100 (23)	35 (19)
Dose reductions				
*N* of patients	57 (72)	22 (68)	63 (80)	23 (70)
*N* of cycles	234 (39)	78 (36)	257 (58)	101 (56)

### Efficacy

**Fig. 2:**
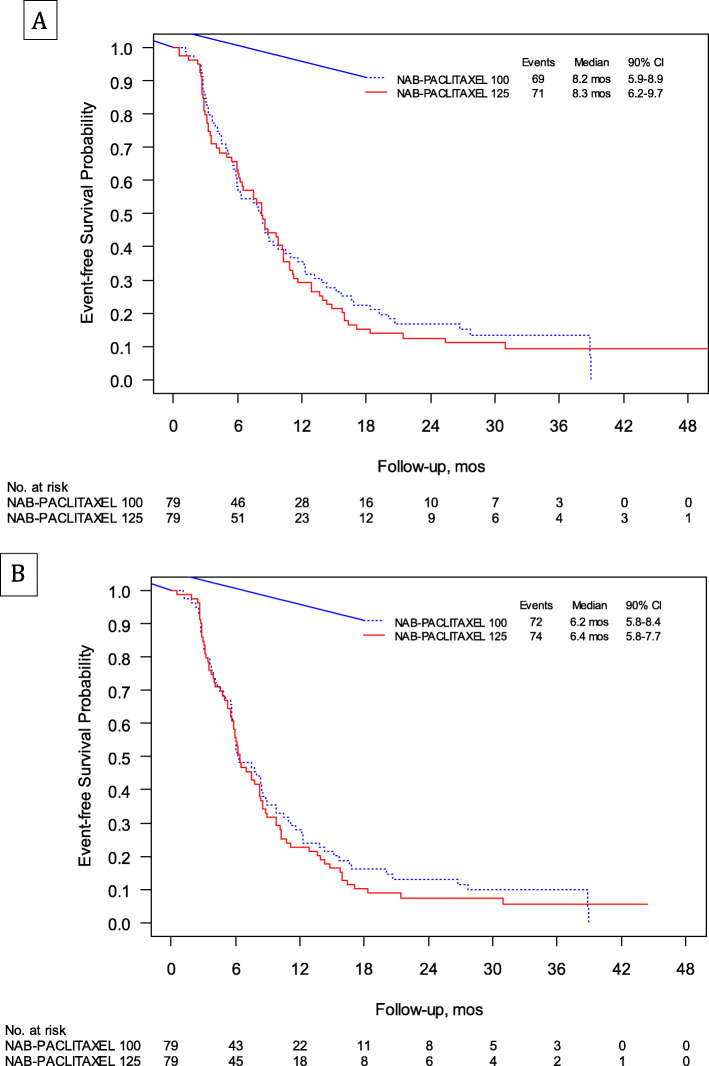
Kaplan-Meier analysis of event-free survival by treatment arm: investigator reported (**a**) and at central review (**b**)

At a median follow-up of 32.6 months (interquartile range, 23.6–41.6), 140 events were observed. The majority of reported events—over 75% in each arm—consisted of PD, with on average only 7% of patients overall experiencing FD (Table 3). The median EFS was 8.2 (90% confidence interval (CI) 5.9–8.9; Brookmeyer-Crowley like test *p* = 0.188) and 8.3 (90% CI 6.2–9.7; Brookmeyer-Crowley like test *p* = 0.078) months for arms A and B, respectively (Fig. 2a). At the central review, FD was identified in 17% of patients on average (Table 3), with a consequent comparative reduction in the median EFS by 2 months in both arms (median EFS 6.2 months vs 6.4 months for arms A and B, respectively) (Fig. 2b). Efficacy outcomes are reported in Table 4.

**Table 3:** Primary endpoint-related events in the overall population and in patients aged 75 years and older. Reported as *N* (%) unless otherwise indicated

Event	Arm A, 100 mg/m^**2**^		Arm B, 125 mg/m^**2**^	
	All (*N* = 79)	Pts aged 75+ (*N* = 33)	All (*N* = 79)	Pts aged 75+ (*N* = 33)
**Investigator reported**				
Pts with reported event	69 (87)	31 (94)	71 (90)	29 (88)
PD	61 (77)	28 (85)	60 (76)	23 (70)
FD	4 (5)	1 (3)	7 (9)	5 (13)
Death	4 (5)	2 (6)	4 (5)	1 (3)
**Central review**				
Pts with reported event	72 (91)	32 (97)	74 (94)	30 (91)
PD	56 (71)	25 (76)	55 (70)	23 (70)
FD	13 (16)	6 (18)	14 (18)	5 (15)
Death	3 (4)	1 (3)	5 (6)	2 (6)

*FD* functional decline, *PD* disease progression, *Pts* patients

**Table 4:** Efficacy outcomes, reported by arm

Event	Arm A, 100 mg/m^**2**^ (***n*** = 79)	Arm B, 125 mg/m^**2**^ (***n*** = 79)
Median EFS, mos (90% CI)	8.2 (5.9–8.9) 6.2 (5.8–8.4)^#^	8.3 (6.2–9.7) 6.4 (5.8–7.7)^#^
Median EFS pts 75+, mos (90% CI)	8.3 (5.7–8.9) 5.9 (5.1–8.3)^#^	8.2 (3.5–10.9) 6.9 (5.5–8.3)^#^
Median PFS, mos (95% CI)	8.3 (5.9–10.5)	8.8 (7.4–10.3)
Median OS, mos (95% CI)	22.4 (17.0–35.6)	20.7 (16.8–28.6)
Best overall response*,* n* (%)	***n*** **= 60**	***n*** **= 65**
CR	4 (7)	1 (2)
PR	20 (33)	26 (40)
SD	24 (40)	18 (28)
PD	9 (15)	14 (21)
NE	3 (5)	6 (9)

*CI* confidence interval, *CR* complete response, *EFS* event-free survival, *mos* months, *NE* not evaluable, *OS* overall survival, *PFS* progression-free survival, *PD* progressive disease, *PR* partial response, *SD* stable disease

^#^Based on central review

*Patients with measurable disease

In patients with measurable disease at baseline (*n* = 125), the ORR was 40% in arm A and 42% in arm B, with a CBR of 80% and 69%, respectively. In those patients who discontinued nab-paclitaxel in the absence of PD, 27% (*n* = 21) in arm A and 34% (*n* = 27) in arm B received a subsequent line of therapy. The median OS in arm A was 22.4 months (95% CI 17.0–35.6) and 20.7 months (95% CI 16.8–28.6) in arm B. The main cause of death in both arms was PD (arm A, *n* = 48 [61%]; arm B, *n* = 45 [57%]).

No difference in the distribution of events was observed within the subgroup of patients aged ≥ 75 years (Table 3). In this group, the median EFS was 8.3 months (90% CI 5.7–8.9) in arm A and 8.2 months (90% CI 3.5–10.9) in arm B. Similar outcomes were observed when EFS was evaluated according to age subgroup (data not shown). The median PFS was 8.3 months (95% CI 5.9–10.5) for arm A and 8.8 months (95% CI 7.4–10.3) for arm B.

### Safety

AEs are reported in Table 5 and are consistent with the known safety profile of nab-paclitaxel in the general population. Myelotoxicity was moderate: one patient in arm A (1%) and three (4%) in arm B experienced G4 neutropenia. G3 febrile neutropaenia was reported in one (1%) and two patients (2.5%) in arms A and B, respectively. The most frequently reported non-haematological AEs were fatigue and peripheral neuropathy. In arm B, G2/3 neurotoxicity occurred more frequently (38% vs 19% in arm A) and with a shorter median time to onset than arm A. In arm A, G2 neurotoxicity was reported after a median of 6.5 cycles (range, 2–11), with G3 reported after a median of 6 cycles (range, 3–9). In contrast, G2 neurotoxicity was reported in arm B after a median of 5 cycles (range, 1–11), with G3 reported after a median of 4.5 cycles (range, 3–8). Severe AEs were reported in ten patients (13%) in each arm. Treatment-related toxicity was reported as the cause of death in three patients: one patient in arm A died due to acute renal failure secondary to diarrhoea; in arm B, one patient died from complications of severe diarrhoea and another from septic shock secondary to *Clostridium difficile* infection after a single dose of weekly nab-paclitaxel, on a background of long-term use of corticosteroids and proton pump inhibitors (PPI).

**Table 5:** Adverse events reported in ≥ 20% of patients if grade 2 or if grades 3–4 occurring at any frequency

Adverse event, ***n*** (%)	Arm A, 100 mg/m^**2**^ (***n*** = 79)				Arm B, 125 mg/m^**2**^ (***n*** = 79)			
	All	Grade 2	Grade 3	Grade 4	All	Grade 2	Grade 3	Grade 4
**Anaemia**	67 (85)	26 (33)	2 (2.5)	–	66 (83.5)	29 (37)	–	–
**Leucopaenia**	47 (59)	20 (25)	7 (9)	–	58 (73)	23 (29)	15 (19)	1 (1)
**Neutropaenia**	46 (58)	18 (23)	14 (18)	1 (1)	55 (67)	13 (16)	25 (32)	3 (4)
**Fatigue**	60 (76)	25 (32)	9 (11)	–	60 (76)	36 (46)	4 (5)	–
**Peripheral neuropathy**	43 (54)	12 (15)	3 (4)	–	51 (64.5)	22 (28)	8 (10)	–
**Nausea/vomiting**	37 (47)	8 (10)	1 (1)	–	30 (38)	12 (15)	2 (2.5)	–
**Alopecia**	35 (44)	24 (30)	/	/	32 (40.5)	21 (27)	/	/
**Myalgia/arthralgia**	33 (42)	11 (14)	1 (1)	–	33 (42)	9 (11)	–	–
**Dyspnoea**	14 (18)	4 (5)	–	–	15 (19)	1 (1)	1 (1)	–
**Fever**	14 (18)	2 (2.5)	–	–	14 (18)	–	1 (1)	–
**Hepatotoxicity**	14 (18)	5 (6)	2 (2.5)	–	16 (20)	1 (1)	1 (1)	–
**Infection**	16 (20)	8 (10)	2 (2.5)	1 (1)	8 (10)°	3 (4)	–	–
**Diarrhoea**	11 (14)	3 (4)	4 (5)	–	15 (19)°	5 (6)	–	–
**Renal toxicity**	2 (2.5)°	–	1 (1)	–	4 (6)	1 (1)	–	–
**Febrile neutropenia**	1 (1)	/	1 (1)	–	2 (2.5)	/	2 (2.5)	–

*CTCAE* Common Terminology Criteria for Adverse Events, / corresponding grade does not exist for this adverse event

°Grade 5 (*n* = 1)

AEs in the patient subgroup aged ≥ 75 years are reported in Additional File 1: Table S1. Of note, two out of the three grade 5 toxicities were registered in this age group. The incidence of grade 2–3 fatigue and neurotoxicity was positively correlated to age in patients treated in arm B, but not observed in arm A (Additional File 2: Table S2).

## Discussion

EFFECT was designed to prospectively identify a dose of nab-paclitaxel that might be safely and efficaciously administered weekly to older women with ABC. Based on the two weekly doses previously evaluated by Gradishar et al. [9] and later explored in a post hoc analysis which concentrated specifically on the older population [11], 150 mg/m^2^ was not explored as it was considered too toxic for older patients. The studied doses of 100 and 125 mg/m^2^ were chosen based on previous results of a phase II study evaluating these two doses in patients with ABC heavily pretreated with taxanes, which showed weekly nab-paclitaxel 100 mg/m^2^ (*n* = 75) had similar antitumour activity and more favourable safety profile than 125 mg/m^2^ (*n* = 106) [16]. Due to the linear pharmacokinetics of nab-paclitaxel, we considered it worthy to investigate both doses in the first-line setting.

Our study showed that the administration of both doses of nab-paclitaxel was feasible, with a comparable median number of delivered cycles in the two arms, but a higher percentage of dose-reduced cycles, and higher rates of treatment discontinuation due to AEs observed in arm B. The two weekly doses of nab-paclitaxel were equally effective and were associated with a similar incidence of FD, noting that the rate of FD increased on the unplanned central review. However, the lower incidence of neurotoxicity observed in arm A makes 100 mg/m^2^ a more feasible dose in older patients with ABC.

The incidence of neurotoxicity and fatigue are integral concerns related to the administration of taxanes. As maintenance of functional status is fundamental to effective care of the elderly, loss of function was included in the EFS evaluation in this study. As such, EFFECT specifically set an elderly-oriented primary endpoint, wherein EFS was calculated based on the occurrence of an event (PD, FD, or death). Central review of data revealed a higher rate of FD than what was initially reported by the investigators, highlighting that FD is commonly under-reported. The explanation for this may be attributed to a lack of familiarity in evaluating FD in clinical trials, as well as FD being a phenomenon that is perhaps under-appreciated in the clinical setting as a whole. It should be noted that due to the restrictive definition set for FD by the study, the increased observation of grade 2–3 fatigue and neurotoxicity reported in arm B did not translate into a higher incidence of FD between the two treatment arms.

As expected, fatigue and peripheral neuropathy were the most frequent non-haematological AEs in both arms. Notably, in arm B, the incidence of grade 2–3 neuropathy was double than that of arm A (19% vs 38%). Contrastingly, at 100 mg/m^2^, the incidence of grade 2 and 3 neurotoxicity was 15% and 4%, respectively. These data are comparable with those reported by Gradishar et al. in an unselected population [9, 10], and equate favourably with those reported by Aapro et al. in patients aged ≥ 65 years, treated at the same dose of weekly nab-paclitaxel (21% grade 3, grade 2 not reported) [11].

In EFFECT, three deaths were reported as the result of AEs. Diarrhoea was associated with the cause of death in all three cases, wherein patients experienced secondary severe dehydration, end-stage renal failure, and septic shock. Chemotherapy-associated diarrhoea represents a significant event which requires close monitoring and aggressive management in older patients, who are at the highest risk of developing severe and fatal complications from associated dehydration, renal insufficiency, electrolyte imbalance, or infection [17, 18]. Age and recent antibiotic exposure are well-recognized risk factors for the development of *Clostridium difficile* infection, as well as the use of PPIs [19] and chemotherapy [20]. This may suggest that the concomitant use of PPIs with chemotherapy be made selectively and cautiously in older patients.

EFFECT evaluated weekly nab-paclitaxel specifically in patients aged ≥ 65 years, in order to address this population’s under-representation in previous clinical trials. However, a representative subgroup of patients aged ≥ 75 years (*n* = 66) allowed the evaluation of this agent in a strictly defined “older” population. Advanced age did not affect treatment feasibility and efficacy but was associated with a higher incidence of both neurotoxicity and fatigue with the 125 mg/m^2^ dose. Notably, two of the three reported deaths occurred in this age subgroup, suggesting a need for closer monitoring of AEs in this potentially less resilient population. The limitations of the unplanned subgroup analysis of patients aged 75 and over are acknowledged, highlighting the need to conduct prospective trials with adequate power to analyse this particular group of patients. Since the conception and completion of EFFECT, data has been published from a small phase II cohort study (*N* = 40) in patients aged 65 and older receiving 100 mg of nab-paclitaxel on days 1, 8, and 15 on a 28-day schedule [21]. Fifty-eight per cent of those patients had treatment-related toxicities at grade 3 or above, with 30% hospitalised as a consequence. This study was conducted in patients who exhibited a higher incidence of markers of vulnerability at baseline geriatric assessment than in the EFFECT population. This disparity might in part explain the higher incidence of toxicity observed in the former study when compared with the 100 mg/m^2^ arm of EFFECT. This further underlines the importance of specifically studying optimal scheduling and dosing in older, vulnerable patient populations.

Reported outcomes in EFFECT were consistent with the first-line single-agent chemotherapy data in older breast cancer patients [22–24]. One of the limitations of this study is that it did not compare the performance of weekly nab-paclitaxel with conventional taxanes, such as solvent-based paclitaxel, which may be seen as the ideal comparator. Two studies have prospectively evaluated paclitaxel 80 mg/m^2^ weekly on days 1, 8, and 15 on a 28-day cycle as the first-line therapy in small groups of older breast cancer patients [19, 20] and found it to be active. However, there were also some significant issues of safety, with one study reporting premature treatment discontinuation in 32% of patients due to fatigue [23] and the other observing toxicity-related treatment interruptions in 15% of patients (5 out of 7 events were due to cardiac toxicity, including two deaths), and grade 2/3 sensory neurotoxicity in 33% of patients [24].

To our knowledge, this is the first prospective trial evaluating nab-paclitaxel in a numerically robust older population that specifically aims to identify the optimal dose for a population known to be at potentially higher risk of treatment-related toxicity. The inclusion of geriatric assessment and the identification of an elderly-related endpoint which included functional decline represent additional strengths of the study. EFS as an endpoint is in line with recommendations generated by the European Organisation for Research and Treatment of Cancer (EORTC) [25], which has advocated for alternative endpoints, such as QoL, toxicity, and functional independence to be considered as a way of improving the clinical design. The findings of EFFECT improve the evidence base for treating older adults with cancer, which is an area of unmet need identified by the American Society of Clinical Oncology [26].

## Conclusions

Weekly taxanes are a suitable treatment option for older patients with MBC. Whilst solvent-based paclitaxel and solvent-based docetaxel are established options, the EFFECT trial has evaluated a role of weekly nab-paclitaxel in this selected population, identifying 100 mg/m^2^ on days 1, 8, and 15 on a 28-day cycle as an effective and well-tolerated dose.

## Supplementary information

**Additional File 1: Supplementary Table 1 (S1).** Distribution of AEs by arm in patients aged ≥75 years. Abbreviations: CTCAE, Common Terminology Criteria for Adverse Events; /, grade does not exist for this adverse event; ° grade 5 *n*=1.

**Additional File 2: Supplementary Table 2 (S2).** Distribution of CTCAE G2-3 fatigue and neurotoxicity by age and arm of treatment. Reported as N (%) unless otherwise indicated. Abbreviations: CTCAE, Common Terminology Criteria for Adverse Events.

## Data Availability

On reasonable request, the data generated and analysed during the current study are available from the corresponding author in accordance with institutional policies.

## Abbreviations

ABC: Advanced breast cancer
ADL: Activity of daily living
AEs: Adverse events
CBR: Clinical benefit rate
CIRS-G: Cumulative Illness Rating Scale–Geriatric
CR: Complete response
EFS: Event-free survival
FD: Functional decline
G: Grade
HR: Hormone receptor
IADL: Instrumental activity of daily living
ORR: Objective response rate
OS: Overall survival
PD: Progressive disease
PFS: Progression-free survival
PR: Partial response
Pts: Patients
SD: Stable disease
