# Supplementary material for: EFFECT: a randomized phase II study of efficacy and impact on function of two doses of nab-paclitaxel as first-line treatment in older women with advanced breast cancer
Source: Breast Cancer Res. 2020 Aug 5;22:83. doi: 10.1186/s13058-020-01319-1 (PMC7405344; doi:10.1186/s13058-020-01319-1)
Supplement: Supplementary file 1 — Additional File 1: Supplementary Table 1 (S1). Distribution of AEs by arm in patients aged ≥75 years. Abbreviations: CTCAE, Common Terminology Criteria for Adverse Events; /, grade does not exist for this adverse event; ° grade 5 n=1. [file 13058_2020_1319_MOESM1_ESM.pdf]

CTCAE grade, n (%)									
	Arm A (n=33)						Arm B (n=33)		
**Adverse Event**	All	2	3	4		All	2	3	4
**Anemia**	29 (88)	13 (39)	1 (3)	-		28 (85)	14 (42)	-	-
**Leucopenia**	21 (64)	10 (30)	2 (6)	-		23 (70)	11 (33)	4 (12)	-
**Neutropenia**	18 (54.5)	6 (18)	7 (21)	-		23 (70)	9 (27)	6 (18)	2 (6)
**Fatigue**	25 (76)	10 (30)	5 (15)	-		27 (82)	18 (55)	2 (6)	-
**Peripheral neuropathy**	15 (45)	3 (9)	2 (6)	-		22 (67)	10 (30)	4 (12)	-
**Nausea/vomiting**	13 (39)	5 (15)	-	-		11 (33)	5 (15)	1 (3)	-
**Alopecia**	13 (39)	8 (24)	/	/		17 (51.5)	8 (24)	/	/
**Myalgia/arthralgia**	14 (42)	5 (15)	1 (3)	-		15 (45)	1 (3)	-	-
**Dyspnoea**	7 (21)	2 (6)	-	-		8 (24)	-	1 (3)	-
**Fever**	5 (15)	1 (3)	-	-		5 (15)	-	1 (3)	-
**Hepatotoxicity**	5 (15)	1 (3)	1 (3)	-		6 (18)	1 (3)	1 (3)	-
**Infection**	3 (10)	-	2 (6)	-		4 (12)	1 (3)	-	-
**Diarrhoea**	4 (12)	2 (6)	2 (6)	-		3 (10)°	1 (3)	-	-
**Renal toxicity**	1 (3)°	-	-	-		1 (3)	-	-	-
**Febrile neutropenia**	1 (3)	/	1 (3)	-		1 (3)	/	1 (3)	-

Supplementary Table 1 (S1): Distribution of AEs by arm in patients aged ≥75 years
Abbreviations: CTCAE, Common Terminology Criteria for Adverse Events; /, grade does not exist for this adverse event; ° grade 5 n=1
