# Supplementary material for: EFFECT: a randomized phase II study of efficacy and impact on function of two doses of nab-paclitaxel as first-line treatment in older women with advanced breast cancer
Source: Breast Cancer Res. 2020 Aug 5;22:83. doi: 10.1186/s13058-020-01319-1 (PMC7405344; doi:10.1186/s13058-020-01319-1)
Supplement: Supplementary file 2 — Additional File 2: Supplementary Table 2 (S2). Distribution of CTCAE G2-3 fatigue and neurotoxicity by age and arm of treatment. Reported as N (%) unless otherwise indicated. Abbreviations: CTCAE, Common Terminology Criteria for Adverse Events. [file 13058_2020_1319_MOESM2_ESM.pdf]

Age	**65-74 years**		**≥ 75 years**	
Arm assignment	Arm A	Arm B	Arm A	Arm B
Number	N=46	N=46	N=33	N=33
Fatigue	19 (41)	20 (43)	15 (45)	20 (61)
Neurotoxicity	10 (22)	16 (35)	5 (15)	14 (42)

Supplementary Table 2 (S2): Distribution of CTCAE G2-3 fatigue and neurotoxicity by age and arm of treatment. Reported as N (%) unless otherwise indicated.
Abbreviations: CTCAE, Common Terminology Criteria for Adverse Events
